# Pulmonary Function Tests in the Evaluation of Early Lung Disease in Cystic Fibrosis

**DOI:** 10.3390/jcm12144735

**Published:** 2023-07-17

**Authors:** Katarzyna Walicka-Serzysko, Magdalena Postek, Urszula Borawska-Kowalczyk, Justyna Milczewska, Dorota Sands

**Affiliations:** 1 Cystic Fibrosis Department, Institute of Mother and Child, 01-211 Warsaw, Poland; magdalena.postek@imid.med.pl (M.P.); urszula.borawska@imid.med.pl (U.B.-K.); justyna.milczewska@szpitaldziekanow.pl (J.M.); dorota.sands@imid.med.pl (D.S.); 2 Cystic Fibrosis Centre, Paediatric Hospital, Dziekanow Lesny, 05-092 Łomianki, Poland

**Keywords:** cystic fibrosis, pulmonary function test, lung clearance index, early lung disease, pulmonary exacerbation, impulse oscillometry, spirometry, HRQOL, CFQ-R

## Abstract

Background: Properly evaluating respiratory system dysfunction is essential in children with cystic fibrosis (CF). This prospective study aimed to assess the course of early lung disease based on multiple breath nitrogen washout (MBNW), impulse oscillometry (IOS), and conventional techniques, such as spirometry and body plethysmography. Methods: Over a 2 year recruitment period, subjects with CF aged 7–18 performed pulmonary function tests (PFTs). Moreover, the nutritional and microbiological status, frequency of pulmonary exacerbations (PExs), and patients’ health-related quality of life (HRQoL) were assessed. Results: The mean age of the children (n = 69) was 14.09 ± 3.26 years; F/M 37/32. Spirometry-based diagnoses of normal lung function (forced expiratory volume in 1 s, FEV_1_ ≥ 90%pred), mild (FEV_1_ 70–89%pred) and moderate (FEV_1_ 40–69%pred) lung diseases were established in 34 (49.3%), 25 (36.2%), and 10 (14.5%) patients, respectively. An elevated lung clearance index (LCI > 6.98) was observed in 85% of the subjects with normal FEV_1_. The presence of *Pseudomonas aeruginosa* infection (n = 16) and the number of PExs treated with IV antibiotics were associated with significantly worse PFT results. Conclusions: MBNW and IOS are more helpful tools than conventional techniques in assessing early lung disease in CF. LCI is a more useful parameter for detecting functional abnormalities than FEV_1_ in school-age children.

## 1. Introduction

Lung disease in cystic fibrosis (CF) develops from the first weeks of life, often without any clinical signs and symptoms [1,2,3,4,5]. Over time, lung function deteriorates and irreversible structural changes occur, such as bronchiectasis, cirrhosis, and pulmonary fibrosis [6,7]. Although many organs and systems are affected by CF, the course of lung disease, in most cases, determines the length and quality of life. Thus, early assessment of respiratory system abnormalities is essential, enabling prompt intervention and delaying the progression of pathological changes [8]. Managing early lung disease and preventing its progression are becoming priorities in CF care [5].

However, assessment is problematic due to certain pulmonary function test (PFT) limitations in the paediatric population. Spirometry is the most commonly used test for monitoring lung disease, which is characterised by relatively low sensitivity in the early stages of CF when less advanced structural abnormalities are present. This characteristic of spirometry is due to the pathological process mainly involving the small airways, which are not detected by standard PFTs [9]. In addition, spirometry and body plethysmography are technically challenging to perform correctly and require good cooperation with patients. It is known that this is very difficult and often impossible to achieve in the paediatric group.

A ground-breaking method of PFT is the multiple breath nitrogen washout (MBNW) technique [10,11]. It is a valuable, non-invasive complement to traditional volumetric tests (spirometry and body plethysmography), and it correlates with the results of imaging studies [12]. MBNW allows the detection of abnormalities in small bronchi (<2 mm in diameter) where early pulmonary changes occur. In this method, nitrogen, a component of atmospheric air, is used as a tracer gas. The test involves gradually flushing out the nitrogen while the individual breathes in 100% medical oxygen. This flushing continues until the nitrogen concentration in the lungs decreases to a level equivalent to 1/40 (2.5%) of its initial concentration. Throughout this phase, the reduction in nitrogen concentration is continuously monitored and recorded in real-time. The lung clearance index (LCI) is the most commonly used parameter in MBNW and represents the number of lung turnovers required to clear the nitrogen gas from the lungs. It has a well-established role in diagnosing and monitoring disease progression in children with CF [10,13] as a marker of ventilation inhomogeneity.

The impulse oscillometry system (IOS), another tidal breathing technique, is also a simple, non-invasive tool that measures respiratory impedance. This test is easy and requires minimal cooperation; therefore, it can be used even in young children and poorly cooperating adults [14,15,16,17]. The IOS enables the assessment of resistance and reactance in both the central and peripheral airways. It provides valuable insights into peripheral airway obstruction and lung elasticity. The technique involves the application of pressure impulses at specific frequencies ranging from 5 to 35 Hz to generate vibrations within the respiratory system. The resulting response of the respiratory tissues is recorded and subjected to mathematical analysis. Similarly to MBNW, IOS reflects peripheral airway impairment, where lung disease begins but is not assessed by spirometry. Therefore, these tests detecting the changes in the small airways have started to be used more frequently in diagnosing and monitoring the earliest stages of CF.

Relying solely on spirometry to monitor the progression of cystic fibrosis (CF) lung disease and evaluate treatment effectiveness is insufficient. Using innovative, increasingly available PFTs (MBNW and IOS) combined with their correlation to conventional tests (spirometry and body plethysmography) can help detect lung disease much earlier. While forced expiratory volume in 1 s (FEV_1_) is an established monitoring tool to guide clinical decision making in advanced CF, the MBNW-derived LCI is a sensitive and valuable parameter for revealing lung function decline in children and is becoming more frequently used in clinical practice [5,18].

The presented project expands on studies analysing the usefulness of PFTs in the CF paediatric population conducted at our Cystic Fibrosis Centre. Its assumptions align with the current strategy for diagnosing and treating CF based on the earliest possible detection of abnormalities for prompt clinical intervention.

This study aimed to assess early lung disease in children with CF aged 7–18 years based on the latest PFTs, such as MBNW and IOS, as well as conventional techniques, such as spirometry and body plethysmography. A multivariate analysis of the mechanisms of early CF lung disease development was carried out based on the PFT results, the microbiological status, and the clinical condition of patients, including the history of pulmonary exacerbations (PExs) in the last year. In addition, the patient’s subjective assessment of health-related functioning and quality of life (HRQOL) was evaluated.

We hypothesised that the latest PFTs, such as MBNW and IOS, would be better tools than conventional techniques, like spirometry and body plethysmography, for assessing lung disease in school-age children with CF.

This study, as a preliminary report, was presented in poster form at the European Cystic Fibrosis Conference in Rotterdam, 8–11 June 2022.

## 2. Materials and Methods

### 2.1. Study Design

This prospective observational study was conducted at the Cystic Fibrosis Centre in Dziekanow Lesny, in accordance with the principles outlined in the Declaration of Helsinki and good clinical practice (Institute of Mother and Child grant No 510-38-09). The local ethics committee approved the trial protocol (No. 22/2021). All patients and their legal guardians provided written informed consent before enrolment in the study.

Between January 2021 and December 2022, we recruited patients aged 7–18 with a confirmed diagnosis of CF based on current diagnostic criteria [19,20,21]. According to the study protocol, we intended to enrol 100 children. The inclusion criteria also consisted of stable clinical condition, sputum production, absence of haemoptysis, and other conditions preventing the ability to perform PFTs. Patients with PExs and those who were not able to perform PFTs were excluded from the study. Microbiological sputum samples were collected during the visit and cultured for bacterial species, including *S. aureus* and *P. aeruginosa*, as well as non-tuberculous mycobacteria (NTM) and fungi. Based on data from our CF Centre, the number of PExs treated with intravenous (IV) and/or oral antibiotics in the last year was analysed. Health-related quality of life was evaluated using the Cystic Fibrosis Questionnaire-Revised (CFQ-R) specific questionnaire.

### 2.2. Pulmonary Function Measurements

After completing their daily airway clearance therapy, patients underwent spirometry, MBNW, IOS, and body plethysmography. All lung function measurements were carried out according to the standard ERS guidelines. Spirometry was performed in all children according to the American Thoracic Society/European Respiratory Society criteria [22,23] using the Jaeger Vyntus IOS (CareFusion, Hochberg, Germany).

The body plethysmography was conducted in a sealed, airtight cabin. At least three repeatable and acceptable manoeuvres were carried out. The plethysmography was performed using Master Screen Body/Diff Jaeger (CareFusion, Hochberg, Germany) in pursuance of the ATS/ERS criteria [23]. Whole-body plethysmography results included spirometry and flow-volume curves.

MBNW was performed using the Exhalyzer D device (EcoMedics AG, Duernten, Switzerland, software version 3.2.0). The test was considered successful if at least two or more (usually three) technically acceptable attempts followed the guidelines of the ERS/ATS consensus statement [24]. All results (FRC and LCI) were expressed as the mean of a minimum of two technically acceptable results obtained during one session.

The Vyntus IOS device (CareFusion, Hochberg, Germany) was used for IOS and spirometry. IOS tests were carried out in a sitting position. They were performed in accordance with the requirements specified in the ATS guidelines [25]. The minimum number of technically acceptable attempts was three.

### 2.3. HRQoL Measurement

The Polish version of the Cystic Fibrosis Questionnaire-Revised (CFQ-R) was used to measure the HRQoL. The CFQ-R is a disease-specific, validated, and widely used instrument [26,27]. It contains both generic and disease-specific domains. Three versions of the CFQ-R were used: the CFQ-R Child 6–11 and CFQ-R Child 12–13, which include 35 items divided into 8 domains, and the CFQ-R Teen/Adult aged ≥ 14 years, which includes 50 items divided into 12 domains. The scores ranged from 0 to 100 in each domain, with higher scores indicating higher HRQOL. Response options included ratings of frequency and difficulty on a 4-point Likert scale (1 = always to 4 = never, 1 = a lot of difficulty to 4 = no difficulty) or true or false responses (1 = very true to 4 = very false). Scoring was computed only if at least half of the questions had been answered within each domain. The domain scores of CFQ-R Child and CFQ-R Teen were combined on account of consultation with the author of the questionnaires. Through the combination process, we lost four domains because the teen/adult version contains more dimensions than the child version. The minimal clinically important difference (MCID) for the CFQ-R respiratory scale is 4.0 points, which is the smallest difference in scores that patients perceive as clinically beneficial to them [28].

### 2.4. Statistical Analysis

Data were analysed using STATISTICA version 13.3 and are shown as median or mean ± SD or as a percentage if not indicated otherwise. The Brown–Forsythe test was used to verify the homogeneity of the variance hypothesis. Depending on the normality of distributions and the homogeneity of variances, statistical tests such as Student’s *t*-test, Mann–Whitney’s test, ANOVA, Welch’s, Kruskal–Wallis, and Spearman correlation were used during statistical analysis.

## 3. Results

### 3.1. Study Group

Over the 2 year recruitment period, from January 2021 to December 2022, 69 CF patients were enrolled in the study. The median age of the patients was 14 years (range: 12–17), with a mean age of 14.09 ± 3.26 years (F/M 37/32) and a BMI of 18.56 ± 2.99 kg/cm^2^. In the study group, there were twenty-six homozygotes of the *F508del* mutation, thirty-four heterozygotes, and nine children with other mutations in the *CFTR* gene. Based on sputum cultures, *Pseudomonas aeruginosa* infection was diagnosed in sixteen patients, MSSA—in fifty-three, *Candida albicans*—in twenty-seven and *Aspergillus fumigatus*—in eight, *Scedosporium apiospermum*—in five. None of the subjects had a positive culture for NTM. The characteristics of the study population and microbiological results are outlined in Table 1.

### 3.2. Correlation of Spirometry with MBNW and IOS

Spirometry results showed a mean value of FEV_1_ 85.72 ± 17.66%pred and forced vital capacity (FVC) 96.04 ± 16.06%pred Spirometry-based diagnosis of normal lung function (FEV_1_ ≥ 90%pred) was established in 34 (49.3%) patients, mild lung disease (FEV_1_ 70–89%pred) in 25 (36.2%), and moderate lung disease (FEV_1_ 40–69%pred) in 10 (14.5%). None of the subjects had severe lung disease with FEV_1_ < 40%pred (Table 2). Airway obstruction (FEV_1_/FVC z-score < −1.96) was observed in 19 patients (28%).

Sixty-three (92.6%) children had an LCI greater than 6.98. Abnormal LCI was observed in all patients with moderate (mean 12.10; range 7.54–15.54) and mild lung disease (mean 10.53; range 7.14–15.04). Twenty-nine patients (85%) with normal FEV_1_ (≥90%pred) had increased LCI (mean 8.97; range 6.25–12.11). There was a significant negative correlation between LCI and FEV_1_ (R_Spearman_ = −0.29, *p* < 0.05).

**Figure 1:**
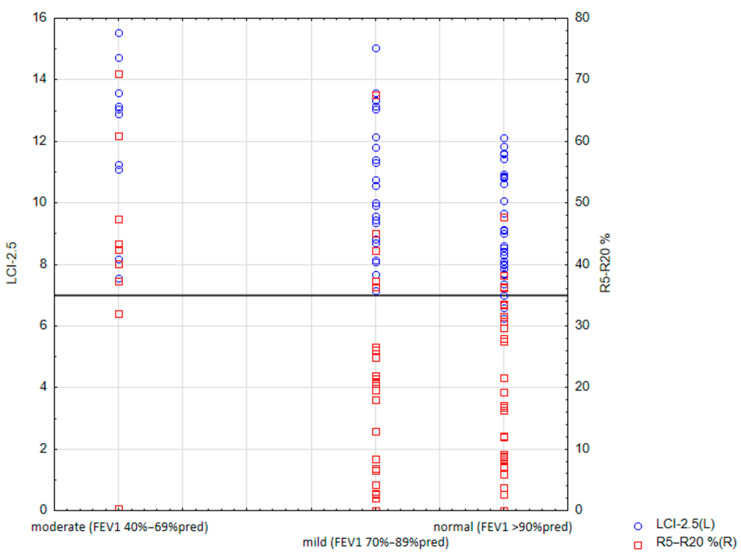
Association of LCI and R5–R20% with FEV_1_ (abbreviations: FEV_1_, forced expiratory volume in the first second; LCI, lung clearance index; R5–R20%, R5, and R20 difference percentage of predicted value).

Patients with moderate lung disease had significantly higher IOS parameters, R5Hz%pred (*p* = 0.016), R20Hz-5Hz%pred (*p* = 0.003), AX (*p* = 0.004), Fres (*p* = 0.011), X20Hz (*p* = 0.02), X20Hz%pred (*p* = 0.02), X5Hz (*p* = 0.004), X5Hz%pred (*p* < 0.001), and MBNW-derived LCI (*p* = 0.003), than in children with normal lung function. The association of LCI and R5–R20% with FEV_1_ is presented in Figure 1. A significant difference in LCI between subjects with mild lung disease and those with normal lung function was also observed (*p* = 0.033).

### 3.3. Correlation of PFTs with a Respiratory Infection

Children with *Pseudomonas aeruginosa* infection (n = 16) had significantly worse PFT results than other participants: lower values of FEV_1_/FVC %pred (*p* = 0.040), FEV_1_/FVC z-score (*p* = 0.037), MEF25% (*p* = 0.021), and MEF25 z-score (*p* = 0.020) in spirometry, and higher values of R at 5 Hz %pred (0.039) in IOS and LCI (*p* = 0.001) in MBNW (Table 3). Infection with MSSA, *Candida albicans*, *Aspergillus fumigatus*, or *Scedosporium apiospermum* did not significantly affect the PFT results.

### 3.4. Correlation of PFTs with Pulmonary Exacerbation

In the study group, two patients did not require treatment due to PEx, thirty-three patients were treated 1–3 times, and thirty-four patients were treated more than three times in the last year (Table 4). Eight children received more than three courses of intravenous antibiotics, twenty-six received 1–3 courses, and thirty-five did not receive such treatment.

The number of PExs treated with IV antibiotics correlated with worse spirometry results, IOS, MBNW, and body plethysmography (*p* < 0.05). No relationship was found for PEx treated orally (Table 5 and Table 6).

Subjects who received more than three intravenous antibiotic courses due to PEx had worse spirometry results (FEV_1_ z-score, FVC%, MEF25%, and MEF25 z-score), IOS (X at 5 Hz %pred), and MBNW (LCI) than patients requiring 0–3 courses (Table 7). Similarly, some PFT parameters (sReff%, sReff z-score, X at 20 Hz %pred, and LCI) were correlated with the total number of PExs (0–3 vs. >3) (Table 8). The differences were statistically significant (*p* < 0.05).

### 3.5. Correlation of PFT Results with HRQoL

The CFQ-R questionnaire allowed a subjective assessment of the health-related quality of life. The highest-rated domain was Physical functioning at 85.2 ± 16.4, while patients evaluated Treatment burden as the lowest at 64.4 ± 16.1 (Table 9).

Results of Physical functioning obtained from the CFQ-R questionnaires correlated positively with spirometry outcomes (FEV_1_%pred, FEV_1_ z-score, MEF25%, and MEF25 z-score) as well as IOS (R at 20 Hz) (Table 10). Additionally, deterioration in lung function measured by IOS (parameters such as R at 20 Hz, R at 5 Hz, and X at 5 Hz) was associated with worse results in the Treatment burden domain. The differences were statistically significant (*p* < 0.05). Such correlations were not found with MBNW or body plethysmography outcomes.

## 4. Discussion

The data presented in this study confirm that MBNW and IOS are more useful tools than conventional techniques like spirometry and body plethysmography in assessing early lung disease in CF. LCI is a more useful parameter for detecting functional abnormalities than FEV_1_ in school-age children and correlates with *Pseudomonas aeruginosa* infection and the number of PExs treated with IV antibiotics.

The trial is part of the current trend of searching for tools to assess early lung disease. In an era of the increasingly widespread use of highly effective CFTR modulator therapy (HEMT), which will modify the course of CF, there is a need for more sensitive PFTs that recognise early functional abnormalities. The ability to detect the evolution of early lung disease and its response to therapy is becoming crucial [5]. New PFTs are being introduced to clinical practice, including MBNW and IOS. They allow for assessing the so-called “quiet zone of the lungs,” i.e., the peripheral part of the respiratory system, the pathological changes of which are not detectable in standard tests such as spirometry or body plethysmography [29]. Together with other techniques, tracking hyperinflation, ventilation heterogeneity, air trapping, and airway obstruction provide an opportunity to assess lung function in school-aged children [6].

This study, conducted in 2021–2022, was meant to include 100 children with CF in a stable condition. Patients who produced sputum to perform microbiological tests and were able to perform PFTs were eligible for the trial. In 2022, a CFTR modulator program was introduced in Poland. Clinical improvement, most of all a significant reduction in cough and mucus production, was observed in patients receiving this treatment after only a few weeks. Therefore, in the second half of 2022, fewer and fewer children with CF under our centre’s care could participate in the study. In total, 69 patients aged 7–18 were included.

As previously reported, MBNW seems to be a more sensitive technique than spirometry for detecting lung function abnormalities in early lung disease in CF [5,30]. Spirometry-based classifications of normal lung function (FEV_1_ ≥ 90%pred) and mild lung disease (FEV_1_ 70–89%pred) failed to detect functional impairment compared to more sensitive technique such as MBNW [5]. Most children (92.6%) in the study group had increased LCI, even those with normal FEV_1_. The majority of patients with normal FEV_1_ (85%) had elevated an LCI (>6.98). Similar to previously conducted research, most subjects who were diagnosed as having normal lung function based on spirometry results were not detected to have significant abnormalities. We can confirm that LCI seems to be a more useful marker than FEV_1_, which detects a higher proportion of pathological changes in children in the early stages of CF lung disease. Between LCI and FEV_1,_ a significant negative correlation (r = −0.29, *p* < 0.05) was observed. As in the other studies, the role of LCI as a more sensitive and useful marker than FEV_1_ for detecting a decline in lung function in school-aged children has been proven [31,32].

Based on the spirometry classification, the IOS and MBNW parameters were associated with lung disease severity. Furthermore, patients with moderate lung disease had significantly higher IOS parameters than patients with normal lung function. A significant difference in LCI between subjects with moderate lung disease and normal lung function, as well as mild lung disease and normal lung function, has also been observed. Therefore, LCI seems to be a more sensitive marker for detecting lung function abnormalities in children with CF than other PFT parameters.

The impact of respiratory infections with pathogenic bacteria and fungi on the course of lung disease has also been demonstrated in many publitions [1,2,8]. In the presented studies, *Pseudomonas aeruginosa* infection was associated with abnormal parameters of PFTs: lower values of FEV_1_/FVC %pred, FEV_1_/FVC z-score, MEF25%, and MEF25 z-score in spirometry, and higher values of R at 5 Hz %pred in IOS and LCI in MBNW. In previous projects, the authors also observed that chronic lung infections, especially *Pseudomonas aeruginosa*, were associated with increased LCI [33,34].

PEx remains one of the main factors that negatively affect clinical outcomes and worsen the prognosis of CF [35,36]. Each event contributes to the risk of permanent lung function decline [37]. The frequency of PExs is closely associated with subsequent progression of lung disease [38,39,40,41]. Even though improvement in FEV_1_ after PEx has been well studied, only a few reports on LCI recovery exist [37,39,42,43]. Perrem et al. showed that a worsening of LCI ≥ 10% increased the odds of failing to recover to baseline lung function in children with acute respiratory symptoms, as measured by LCI and FEV_1_ [44].

Every PEx may negatively affect pulmonary function. Stanojevic et al. described the trajectories of LCI in early school-age years and identified that treatment with either oral or IV antibiotics was associated with higher LCI [45]. In our study, the number of PExs treated with IV antibiotics correlated with worse spirometry, IOS, MBNW, and body plethysmography (*p* < 0.05) results. There was no such relationship found for PExs treated orally.

Subjects who received more than three IV antibiotic courses due to PEx had worse parameters of spirometry (FEV_1_ z-score, FVC%, MEF25%, and MEF25 z-score), IOS (X at 5 Hz %pred), and MBNW (LCI) than patients requiring 0–3 courses (Table 7). Similarly, some PFT parameters (sReff%, sReff z-score, Xat20Hz%pred, and LCI) were correlated with the total number of PExs (0–3 vs. >3) (Table 8). Some studies also reported that both IOS and MBNW measurements are useful in evaluating paediatric patients with CF during PExs or in stable conditions [46,47].

The relationship between HRQOL and lung function in children and adults with CF was previously studied. Moderate positive cross-sectional associations between FEV_1_ and the CFQ-R Respiratory and Physical domains have been reported in children and adults with CF [48,49]. Still, longitudinal trials have shown inconsistent results [50,51]. Studies comparing LCI and CFQ-R scores in children with CF have also been contradictory [52,53,54,55].

In our research, the PFT results suggest that in early lung disease, CF spirometry parameters, to a greater extent, reflect the physical functioning of school-aged children. Higher values of FEV_1_/FVC%, FEV_1_/FVC z-score, MEF25%, and MEF25 z-score correlated with better subjective assessment of physical functioning in the CFQ-R questionnaire. An increase in resistance in the central airways in IOS was associated with a worse assessment of the functioning of the respiratory system and more significant limitations related to treatment in the CFQ-R. On the other hand, the high sensitivity of LCI associated with the inhomogeneity of ventilation allows for detecting emerging changes early enough that they do not affect the assessment of physical functioning or the treatment burden.

The strength of this study is that MBNW and IOS were performed along with conventional tests, such as spirometry and body plethysmography, in school-aged children with CF. These techniques are usually not carried out together due to some limitations for wider use, particularly the skills and time it takes to obtain reliable manoeuvres in the paediatric population. The authors are aware of several study limitations. Due to a small group of subjects enrolled, the results cannot be generalised to the whole population of children with CF. This project was accepted and conducted before the widespread introduction of CFTR modulators in Poland. It is known that HEMT may impact the magnitude of PFT outcomes. In an era of increasing access to HEMT, the ability to detect the evolution of early disease will be increasingly important. Currently, after introducing CFTR modulator therapy in Poland, we plan to continue this study with the same patient cohort.

## 5. Conclusions

The present study supports using MBNW and IOS as more sensitive functional tools than conventional techniques for detecting and monitoring early lung disease in school-age children with CF. IOS, as an alternative method, can be a complement to MBNW. The ease of using those methods by the patient, no need for full cooperation, as opposed to spirometry and body plethysmography, significantly affect their wide application. Spirometry-based classifications of normal lung function and mild lung disease caused the failure of FEV_1_ to detect significant abnormalities. LCI is a useful parameter that captures a higher proportion of pathological changes in patients with a decline in lung function than FEV_1_. It correlates with *Pseudomonas aeruginosa* infection and the number of PExs treated with IV antibiotics. The high sensitivity of this parameter may allow for detecting emerging changes early enough that they do not affect the subjective evaluation of physical functioning or the treatment burden.

Further studies with new PFTs are needed to monitor the evolution of early lung disease and its response to therapy, specifically in children who receive HEMT.

## Figures and Tables

**Table 1:** Baseline characteristics of the study group (abbreviations: BMI, body mass index; FEV_1_, forced expiratory volume in the first second; and LCI, lung clearance index).

Number of Patients	69
Age in years, mean ± SD	14.09 ± 3.26
Male/female	32/37
Weight z-score	−0.09 ± 0.82
Height z-score	0.25 ± 0.88
BMI, mean ± SD	18.56 ± 2.99
BMI z-score mean ± SD	−0.31 ± 0.80
*F508del* homozygous, n (%)	26 (38%)
*F508del* heterozygous	34 (49%)
Other/other	9 (13%)
MSSA infection	53 (77%)
*Pseudomonas aeruginosa* infection	16 (23%)
*Aspergillus fumigatus* infection	8 (12%)
*Scedosporium apiospermum* infection	5 (7%)
FEV_1_ z-score < −1.64	23 (33%)
FEV_1_ z-score, mean ± SD	−1.15 ± 1.5
FEV_1_ %predicted, mean ± SD	85.72 ± 17.66
LCI 2.5%, mean ± SD	9.86 ± 2.50

**Table 2:** Correlation of spirometry with MBNW and IOS parameters (abbreviations: MBNW, multiple breath nitrogen washout; IOS, impulse oscillometry; FEV_1_, forced expiratory volume in the first second; FVC, forced vital capacity; LCI, lung clearance index; R5Hz, resistance at 5Hz; AX, area of reactance; Fres, resonance frequency; and X20Hz, reactance at 20 Hz).

		MBNW Parameters		IOS Parameters							
FEV_1_	N (%)	LCI > 6.98 N (%)	LCI Mean [Range]	R5Hz%Pred Mean ± SD	R5Hz-20Hz%Pred Mean ± SD	AX Mean ± SD	Fres Mean ± SD	X20Hz Mean ± SD	X20Hz%Pred Mean ± SD	X5Hz Mean ± SD	X5Hz%Pred Mean ± SD
FEV_1_ ≥ 90%pred	34 (49.3)	29 (85)	8.97; [6.25–12.11]	92.57 ± 36.11	17.32 ± 13.68	0.64 ± 0.52	13.75 ± 4.58	0.07 ± 0.05	111.33 ± 91.89	−0.15 ± 0.076	115.33 ± 50.98
FEV_1_ 70–89%pred	25 (36.2)	25 (100)	10.53; [7.14–15.04]	110.17 ± 44.59	20.66 ± 16.75	0.82 ± 0.97	14.67 ± 5.30	0.05 ± 0.06	66.74 ± 96.52	−0.15 ± 0.11	150.65 ± 91.61
FEV_1_ 40–69%pred	10 (14.5)	10 (100)	12.10; [7.54–15.54]	129.33 ± 49.16	41.65 ± 19.66	1.71 ± 0.94	19.58 ± 4.70	0.001 ± 0.06	−12.56 ± 118.43	−0.28 ± 0.10	240.11 ± 128.29
FEV_1_ < 40%pred	-	-	-	-	-	-	-	-	-	-	-
FEV_1_/FVC z-score < −1.96	19 (28)	-	-	-	-	-	-	-	-	-	-
p FEV_1_ ≥ 90%pred vs. FEV_1_ 40–69%pred	-	-	0.003	0.016	0.003	0.004	0.011	0.02	0.02	0.004	< 0.001
p FEV_1_ ≥ 90%pred vs FEV_1_ 70–89%pred	-	-	0.033	-	-	-	-	-	-	-	-
LCI > 6.98	63 (92.6)	-	-	-	-	-	-	-	-	-	-

**Table 3:** Correlation of pulmonary function test (PFT) results with *Pseudomonas aeruginosa* infection in the study group (abbreviations: FEV_1_, forced expiratory volume in the first second; FVC, forced vital capacity; MEF25, maximum expiratory flow at 25% of the forced vital capacity; R at 5 Hz, resistance at 5 Hz; and LCI, lung clearance index).

Parameter	R_Spearmana_ (*p* < 0.05)
FEV_1_/FVC %pred	0.040
FEV_1_/FVC z-score	0.037
MEF25%	0.021
MEF25 z-score	0.020
R at 5 Hz %pred	0.039
LCI-2.5	0.001

**Table 4:** Groups of patients depending on the number of PExs treated with intravenous or/and oral antibiotics in the last year. PEx, pulmonary exacerbation.

	0 PEx	1–3 PExs	>3 PExs
PEx oral	2	45	22
PEx iv	35	26	8
PEx oral + iv	2	33	34

**Table 5:** Correlation of spirometry, IOS, and MBNW results with the number of PExs treated with intravenous antibiotics (abbreviations: IOS, impulse oscillometry; MBNW, multiple breath nitrogen washout; PEx pulmonary exacerbation; FEV_1_, forced expiratory volume in the first second; FVC, forced vital capacity; MEF25, maximum expiratory flow at 25% of the forced vital capacity; MEF50, maximum expiratory flow at 50% of the forced vital capacity; X at 20 Hz, reactance at 20 Hz; X at 5 Hz, reactance at 5 Hz; and LCI, lung clearance index).

Parameter	FEV_1_ %pred	FEV_1_ z-Score	FVC %pred	FEV1/FVC %pred	FEV1/FVC z-Score	MEF_25_ %pred	MEF_25_ z-Score	MEF_50_ %pred	MEF_50_ z-Score	X at 20 Hz %pred	X at 5 Hz %pred	LCI-2.5
R_spearmana_ (*p* < 0.05)	−0.38	−0.39	−0.28	−0.31	−0.32	−0.42	−0.42	−0.38	−0.37	−0.26	0.34	0.57

**Table 6:** Correlation of body plethysmography results with the number of PExs treated with oral and intravenous antibiotics (abbreviations: PEx, pulmonary exacerbation; Rtot, total airway resistance; sReff, effective specific airway resistance; FRC, functional residual capacity; TLC, total lung capacity; and RV, residual volume).

Group/R_Spearman_	Rtot	Rtot z-Score	sReff %pred	sReff z-Score	Reff	Reff %pred	Reff z-Score	FRC %pred	FRC z-Score	TLC %pred	TLC z-Score	RV%TLC	RV/TLC %pred	RV/TLC z-Score
PEx oral	−0.03	0.03	0.14	0.17	0.04	0.10	0.12	0.23	0.24	0.17	0.16	0.22	0.21	0.20
PEx iv	0.27 *	0.26 *	0.36 *	0.41 *	0.32 *	0.27 *	0.31 *	0.24	0.21	0.22	0.21	0.38 *	0.42 *	0.34 *

* R_Spearman_ for *p* < 0.05.

**Table 7:** Correlation of PFT results with the number of intravenous antibiotic therapies due to pulmonary exacerbation (PEx 0–3 vs. PEx > 3). (Abbreviations: PFT, pulmonary function test; PEx, pulmonary exacerbation FEV_1_, forced expiratory volume in the first second; FVC, forced vital capacity; MEF25, maximum expiratory flow at 25% of the forced vital capacity; X at 5 Hz, reactance at 5 Hz; and LCI, lung clearance index.)

Parameter	*p* 0–3 PEx iv vs. >3 PEx iv
FEV1 z-score	0.044
FVC %pred	0.049
MEF25 %pred	0.031
MEF25 z-score	0.030
X at 5 Hz %pred	0.047
LCI-2.5	<0.001

Concentrations of T1AM and TA1 in medium and cell lysates after 0, 5, 15, 30, and 60 min of infusion. Data represent mean ± SEM, n = 3 per group, and are expressed as nM. T1AM or TA1 contents were measured in medium and lysate HMC3 cells, which were incubated for 0, 5, 15, 30, and 60 min with T1AM (0.1 µM). N.D., not detectable.

**Table 8:** Correlation of PFT results with the total number of pulmonary exacerbations (PEx 0–3 vs. PEx > 3). Abbreviations: PFT, pulmonary function test; PEx pulmonary exacerbation; sReff, effective specific airway resistance; X at 20 Hz, reactance at 20 Hz; LCI, lung clearance index.

Parameter	*p* 0–3 PExs vs. >3 PExs
sReff %	0.020
sReff z-score	0.011
X at 20 Hz %pred	0.040
LCI-2.5	0.001

**Table 9:** Results of the CFQ-R questionnaire in the study group. CFQ-R, Cystic Fibrosis Questionnaire-Revised.

	CFQ-R							
	Physical	Emotional State	Social	Body Imagine	Eating	Treatment Burden	Respiratory	Digestion
M ± SD	85.31 ± 16.4	74.14 ± 15.5	67.82 ± 18.2	74.88 ± 24.1	79.39 ± 22.9	64.4 ± 16.1	77.86 ± 16.4	83.09 ± 17.4
MIN	38.89	26.67	19.05	0	0	33.33	22.22	33.33
MAX	100	100	100	100	100	100	100	100

**Table 10:** Correlation of PFTs’ results with the CFQ-R questionnaires’ results (abbreviations: PFTs, pulmonary function tests; CFQ-R, Cystic Fibrosis Questionnaire-Revised; FEV_1_, forced expiratory volume in the first second; FVC, forced vital capacity; MEF25, maximum expiratory flow at 25% of the forced vital capacity; R at 20 Hz, resistance at 20 Hz; R at 5 Hz, resistance at 5 Hz; and X at 5 Hz, reactance at 5 Hz).

CFQ-R	Parameter	R_Spearmana_ (*p* < 0.05)
Physical	FEV_1_/FVC %pred	0.28
	FEV_1_/FVC z-score	0.28
	MEF_25%pred_	0.24
	MEF_25_ z-score	0.24
Respiratory	R at 20 Hz	−0.25
Treatment burden	R at 20 Hz	0.32
	R at 5 Hz	0.32
	X at 5 Hz	−0.3

## Data Availability

The data presented in this study are available on request from the corresponding author.
